# Construction of a molecular clone of ovine enzootic nasal tumor virus

**DOI:** 10.1186/s12985-016-0660-x

**Published:** 2016-12-30

**Authors:** Scott R. Walsh, María Carla Rosales Gerpe, Sarah K. Wootton

**Affiliations:** 1 Department of Pathobiology, Ontario Veterinary College, University of Guelph, Guelph, Ontario Canada; 2 McMaster Immunology Research Centre, McMaster University, Hamilton, Ontario Canada

## Abstract

**Background:**

Enzootic nasal tumor virus (ENTV-1) is an ovine betaretrovirus that has been linked to enzootic nasal adenocarcinoma (ENA), a contagious tumor of the ethmoid turbinates of sheep. Transmission experiments performed using virus isolated from cell free nasal tumor homogenates suggest that ENTV-1 is the causative agent of ENA; however, this etiological relationship has not been conclusively proven due to the fact that the virus cannot be propagated in vitro nor is there an infectious molecular clone of the virus.

**Methods:**

Here we report construction of a molecular clone of ENTV-1 and demonstrate that transfection of this molecular clone into HEK 293T cells produces mature virus particles.

**Results:**

Analysis of recombinant virus particles derived from the initial molecular clone revealed a defect in the proteolytic processing of Gag; however, this defect could be corrected by co-expression of the Gag-Pro-Pol polyprotein from the highly related Jaagsiekte sheep retrovirus (JSRV) suggesting that the polyprotein cleavage sites in the ENTV-1 molecular clone were functional. Mutagenesis of the molecular clone to correct amino acid variants identified within the *pro* gene did not restore proteolytic processing; whereas deletion of one proline residue from a polyproline tract located in variable region 1 (VR1) of the matrix resulted in production of CA protein of the mature (cleaved) size strongly suggesting that normal virion morphogenesis and polyprotein cleavage took place. Finally, electron microscopy revealed the presence of spherical virus particles with an eccentric capsid and an average diameter of about 100 nm.

**Conclusion:**

In summary, we have constructed the first molecular clone of ENTV-1 from which mature virus particles can be produced. Future experiments using virus produced from this molecular clone can now be conducted to fulfill Koch’s postulates and demonstrate that ENTV-1 is necessary and sufficient to induce ENA in sheep.

## Background

Protease-mediated processing of the Gag-Pro-Pol polyprotein is an essential step in the replication of retroviruses [1]. The protease is activated concurrent with egress, or shortly thereafter, at which time it is released from the polyprotein via an autocatalytic reaction followed by proteolytic processing of the remainder of the polyprotein [2]. The mechanism or trigger for activation of retroviral proteases is unclear. Strict regulation of the protease is required to prevent premature activation, which would inhibit virus assembly, budding and infectivity [3–6]. This is especially important for betaretroviruses as the core assembles within an intracellular compartment before transport to the plasma membrane and release [2]. Several studies have demonstrated that protein conformation/dimerization [4, 7–9] and oxidation [10, 11] play an integral role in protease activation, but activation of ovine betaretrovirus proteolytic processing has not been studied.

Inactive protease is detrimental to retroviral replication because in the absence of protease processing the virion will not convert to the mature metastable conformation required for the virus to become infectious. Indeed, there is a class of antiretroviral drugs designed to specifically inhibit the protease and consequently inhibit virus replication [12, 13].

Enzootic nasal tumor virus (ENTV)-1 is an ovine betaretrovirus that is associated with enzootic nasal adenocarcinoma (ENA), a nasal tumor of sheep [14]. Experiments showing transmission of ENA to a healthy lamb by inoculation with cell-free ENA tumor homogenate containing ENTV-1 antigens suggested that ENTV-1 is the causative agent of ENA, but did not completely fulfil Koch’s postulates [14]. A factor limiting these experiments is an inability to produce high titer virus from cell culture since the virus cannot be propagated in vitro. In the study presented here, we sought to resolve this issue by constructing a molecular clone from which infectious ENTV-1 could be generated. Transfection of HEK 293T cells with the ENTV-1 molecular clone led to the production of virus particles, but processing of the Gag polyprotein was not observed. The protease could not be activated by treatment with a reducing agent but could be complemented with the JSRV Gag-Pro-Pol polyprotein producing virus particles with fully processed Gag. Mutagenesis of non-conserved amino acids within the protease domain failed to restore Gag polyprotein processing; however, removal of an additional proline residue from a polyproline tract in the matrix protein resulted in the production of fully mature virus particles. This is the first report demonstrating that ENTV-1 can be produced from a molecular clone providing the foundation for further studies investigating the pathogenesis and determinants of tissue tropism of this poorly understood virus.

## Methods

### Cloning and vector construction

Two overlapping fragments comprising the ENTV-1 genome were amplified from genomic DNA (Genbank accession number FJ744146) isolated from an ENA sample using PfuUltra II Fusion HS DNA Polymerase (Agilent Technologies, Missisauga, Ontario, Canada) and the E5F/E5R and E3F/E3R primer pairs (Table 1) for the 5’ fragment and the 3’ fragment, respectively. PCR products were cloned using the TOPO TA cloning kit (Life Technologies). Subsequently, the cytomegalovirus (CMV) promoter was amplified from the pcDNA3.1 plasmid and fused to the 5’ ENTV-1 genome fragment at the 5’ border of the R region using overlap extension PCR (using primers CMVF, RCMVR, E5F and EGagR, Table 1). The CMV-RU5 fusion fragment was inserted into the 5’ ENTV-1 fragment using XbaI and BsrGI restriction sites. The 5’ fragment was excised using XbaI and MmeI and the 3’ fragment was excised using NotI and MmeI and both were ligated into

**Table 1:** Primers used in cloning and mutagenesis

Primer Name	Sequence 5’ to 3’
E5F	AGCAAGGATCAGCCATTCT
E5R	GAACACAGATAAAGGGAGGC
E3F	TAACACCCAACTAGTAAAGCTG
E3R	GGCTGATACCTTGCTTTATTG
CMVF	GCTCTAGACGTTACATAACTTACGGTAAATGGCC
RCMVR	AGAATGGCTGATACCTTGCCGGAGGCTGGATCGGTC
EGagR	TCCCCTACCTTTTTCCATGTT
JEenvR	ATTTCTCTATCCCATCCTAAGG
EU5F	CCCATCTTTTGCCTCTCTTAT
EenvR	CTCCGTTTTGTATCCGTTGC
JU5F	CCCCATCTTTTGTCTCTCTCTT
JenvR	CCCATTTTGTACCCGCTGT
EproS199P F	GGCCTACTACATGGCCAAAACAGACGGCTATTTCC
EproS199P R	AAATAGCCGTCTGTTTTGGCCATGTAGTAGGCCAAT
EproT202M F	ACATGGCCAAAACAGATGGCTATTTCCACTCT
EproT202M R	GAGTGGAAATAGCCATCTGTTTTGGCCATGTA
EproT289I F	AAACAACATCAAGGCATCATTTTGCCCCTTGAT
EproT289I R	ATCAAGGGGCAAAATGATGCCTTGATGTTGTTT

Primers utilized in the generation and mutagenesis of the molecular clone and in screening for ENTV-1 transcripts derived from the molecular clone are listed along with the corresponding sequences on the right (5’ to 3’)

XbaI and NotI digested low copy number plasmid, pLG338/30 (provided by Dr. Ronald C. Montelaro through the AIDS Reagent Program, Bethesda, Maryland, USA) [15]. Plasmids containing ENTV-1 genome fragments were prone to recombination in *E. coli* and thus were incubated at a reduced temperature (30 °C). The JSRV molecular clone, pCMVJSRV21 [16], and the JSRV Gag-Pro-Pol polyprotein expression vector, pCMVGPP-MX-4CTE [17], were kind gifts from Dr. Massimo Palmarini, University of Glasgow, Scotland.

Amino acid changes were introduced into the ENTV-1 molecular clone (Fig. 3b) using a modified site-directed mutagenesis protocol as outlined by Wang et al. [18] using the primer pairs (EproS199P F/R, EproT202M F/R and EproT289I F/R) in Table 1 and the KOD Hot Start DNA Polymerase kit (Novagen) according to the manufacturer’s instructions. Briefly, mutagenesis was performed with two separate amplification reactions containing either forward or reverse primers to amplify the molecular clone plasmid for three rounds. The two single-primer PCR products were combined in one tube and amplified for a further 14 rounds. Methylated parental plasmid DNA was digested with DpnI before transformation into competent GT116 (InvivoGen) *E. coli*.

### RT-PCR

**Fig. 1:**
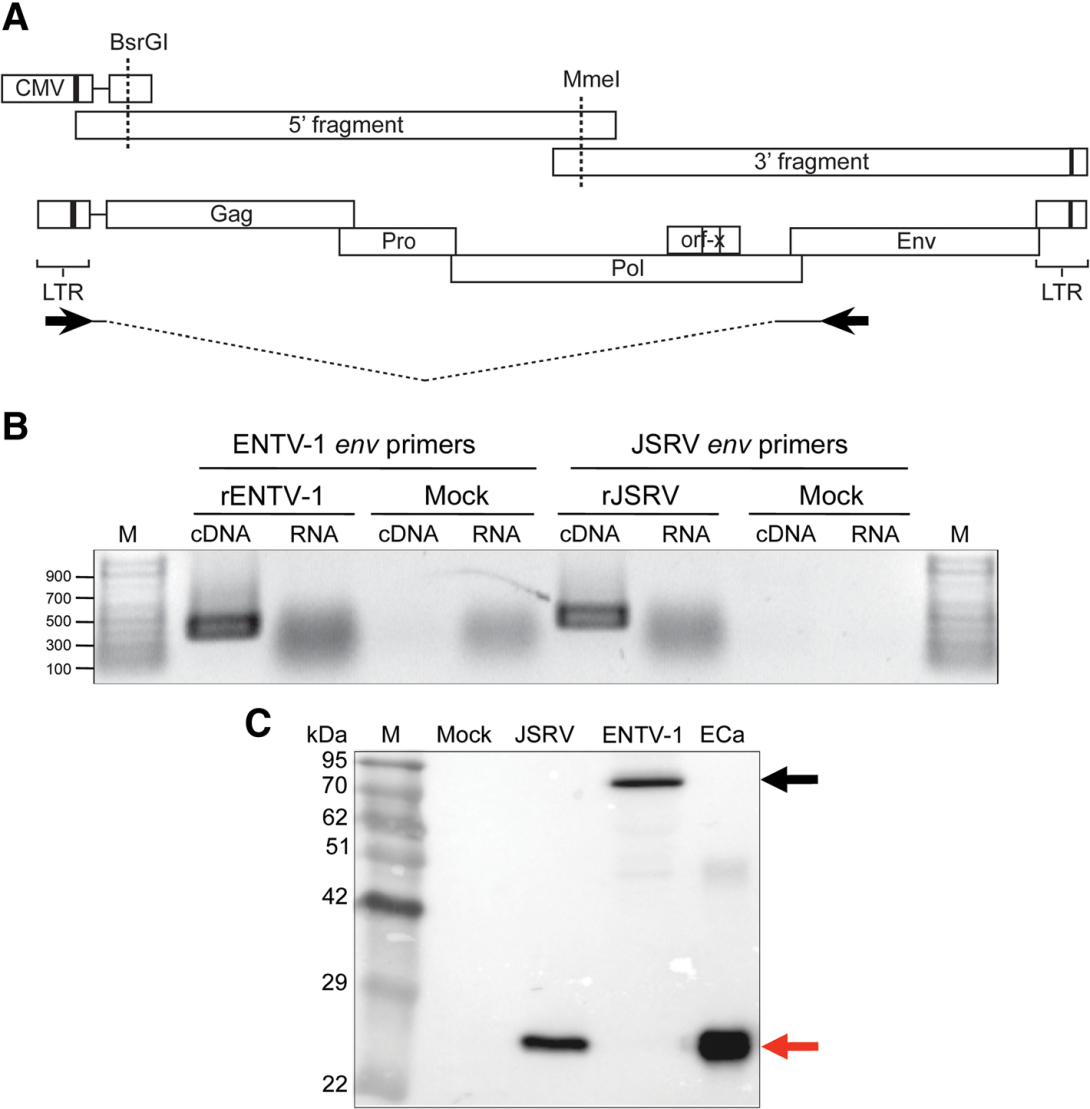
Lack of Gag-Pro-Pol polyprotein processing in virions derived from the original ENTV-1 molecular clone. **a** The ENTV-1 genome was amplified from genomic DNA isolated from an ENA tumor (ENTV-1NA4) in two overlapping fragments. The CMV promoter was fused to the 5’ ENTV-1 genome at the 5’ border of the R region in an intermediate fragment by overlap extension PCR and was introduced into the 5’ genome fragment via the BsrGI restriction site. The fragments were ligated together at the MmeI restriction site and the ENTV-1 molecular clone was generated by ligation into the pLG338/30 plasmid. **b** Singly spliced transcripts coding for the envelope protein were detected in RNA extracted from HEK 293T cells transfected with the ENTV-1 or JSRV molecular clone and the resulting PCR products are shown. A schematic outlining this strategy and the location of the primers (*arrows*) used in the RT-PCR is shown above. *M* indicates the lanes containing a 100 bp DNA ladder (Life Technologies). **c** Immunoblot analysis of capsid protein in the lysates of concentrated virus particles derived from the indicated molecular clones after transfection of HEK 293T cells. *M* indicates the lane containing the molecular weight marker and lanes labelled ECa contain bacterially expressed and purified ENTV-1 capsid protein. The *black arrows* indicate unprocessed Gag at 78 kDa and the *red arrows* indicate processed capsid at 26 kDa. The dashes on the right hand side demarcate unprocessed forms of Gag

Total RNA was extracted from HEK 293T cells transfected with either pCMVENTV-1 or pCMVJSRV21 and cDNA was generated using the JEenvR primer (Table 1) and the SuperScript II Reverse Transcriptase kit (Life Technologies) according to manufacturer instructions. A PCR product representing the spliced envelope transcript from JSRV (393 bp) and ENTV (384 bp) were amplified from the cDNA using Platinum PCR SuperMix (Life Technologies) and the JU5F/JenvR and EU5F/EenvR primer pairs (sequences shown in Table 1 and genome location shown in Fig. 1a) to amplify JSRV and ENTV-1 mRNA transcripts, respectively.

### Cell lines and virus production

HEK 293T, GSM-T (a goat synovial membrane cell line immortalized with telomerase expression; a kind gift from Dr. Yahia Chebloune, Université Joseph Fourier), SSF (sheep skin fibroblasts) and ONTC (ovine nasal tumor cell line) [19] cells were grown in Dulbecco’s minimum essential medium (DMEM) supplemented with 10% fetal bovine serum and 2 mM glutamine at 37 °C under 5% CO_2_. To produce primary sheep respiratory cells, fetal lung and nasal tissue was diced with a scalpel and subsequently dispersed by passage through a 20-gauge needle. Non-adherent cells were removed 72 h after plating and the remaining adherent cells were treated with trypsin for 3 minutes to remove fibroblast cells and enrich for epithelial cells. The cells were propagated in epithelial specific medium (KSFM; Gibco) for three to four passages before transfection.

For virus production, 5 × 10^6^ HEK 293T cells plated on 10 cm dishes were transfected with either the JSRV or ENTV molecular clone using the calcium phosphate co-precipitation method and medium was replaced after 16 h with 5 ml of fresh medium. The virus-containing medium was harvested 48 h later, passed through a 0.45 μm filter and pelleted by ultracentrifugation through a 20% sucrose cushion in a SW32 Ti rotor (Beckman Coulter Canada, Mississauga, Ontario, Canada) at 60,000 × g for 2 h at 4 °C. The viral pellet was suspended in TNE buffer [16].

### Western blot analysis

Western blotting was conducted as described previously [20] using monoclonal antibodies specific for the envelope protein of ovine betaretroviruses [21] (i.e., JSRV, ENTV and enJSRV) and the capsid protein [22] (kindly provided by Dr. Hung Fan, University of California, Irvine).

### Electron microscopy of ENTV- 1 particles

Five microliters of ENTV-1 virus purified through a 20% sucrose cushion were aliquoted onto a glow discharged, 200 mesh, copper grid (Electron Microscopy Sciences), with formvar and carbon coating. The droplet was wicked off with filter paper after 30 s and the grid floated on a droplet of 2% Uranyl Acetate for 10 s. The sample was imaged in the FEI Tecnai G2 FEG (Microscopy Imaging Facility, University of Guelph) at 200 kV and images collected with the Gatan 4 K bottom mount CCD camera using the Gatan Digital Micrograph software.

## Results

### The ENTV-1 molecular clone produces unprocessed immature virions

ENTV-1 cannot be propagated in conventional cell culture systems, therefore it was necessary to generate a molecular clone in order to produce virus for experimental purposes and to show that ENTV-1 alone is sufficient to induce nasal tumors in sheep. The full-length ENTV-1 genome was amplified from genomic DNA extracted from ENA tumor tissue in two overlapping fragments and cloned into a plasmid with the CMV promoter fused to the 5’ end of the R region (Fig. 1a). The 5’ U3 was replaced with a CMV promoter to promote high-level expression in human cells. The resulting plasmid (pCMVENTV-1) was transfected into HEK 293T cells and after 48 h, RNA was extracted and subjected to RT-PCR using a forward primer in the U5 region and a reverse primer in the signal peptide region of *env* (see black arrows in Fig. 1a) producing a band at approximately 384 bp (Fig. 1b). This band represented spliced ENTV-1 envelope transcripts and demonstrated that the molecular clone could support transcription of viral genes. The JSRV molecular clone was analysed in parallel and showed a PCR product of the predicted 393 bp (Fig. 1b). Recombinant ENTV-1 and JSRV particles produced in HEK 293T and concentrated by ultracentrifugation were subjected to SDS-PAGE separation and immunoblot analysis using a monoclonal antibody against the JSRV capsid, which cross-reacts with the ENTV-1 capsid. A protein of 26 kDa was detected in the lane containing JSRV lysate (Fig. 1c, lane 2) as well as in the lane containing bacterially expressed ENTV-1 capsid (ECa) protein (Fig. 1c, lane 4). However, this 26 kDa protein was absent in the ENTV-1 molecular clone (Fig. 1c, lane 3) and instead, a protein of approximately 78 kDa, which is the expected size of the unprocessed Gag polyprotein, was detected. Taken together, these results suggest that the ENTV-1 molecular clone is able to direct production of virus particles but that there is a defect in proteolytic processing.

### The JSRV Gag-Pro-Pol polyprotein can complement and correct the processing defect of the ENTV-1 molecular clone

**Fig. 2:**
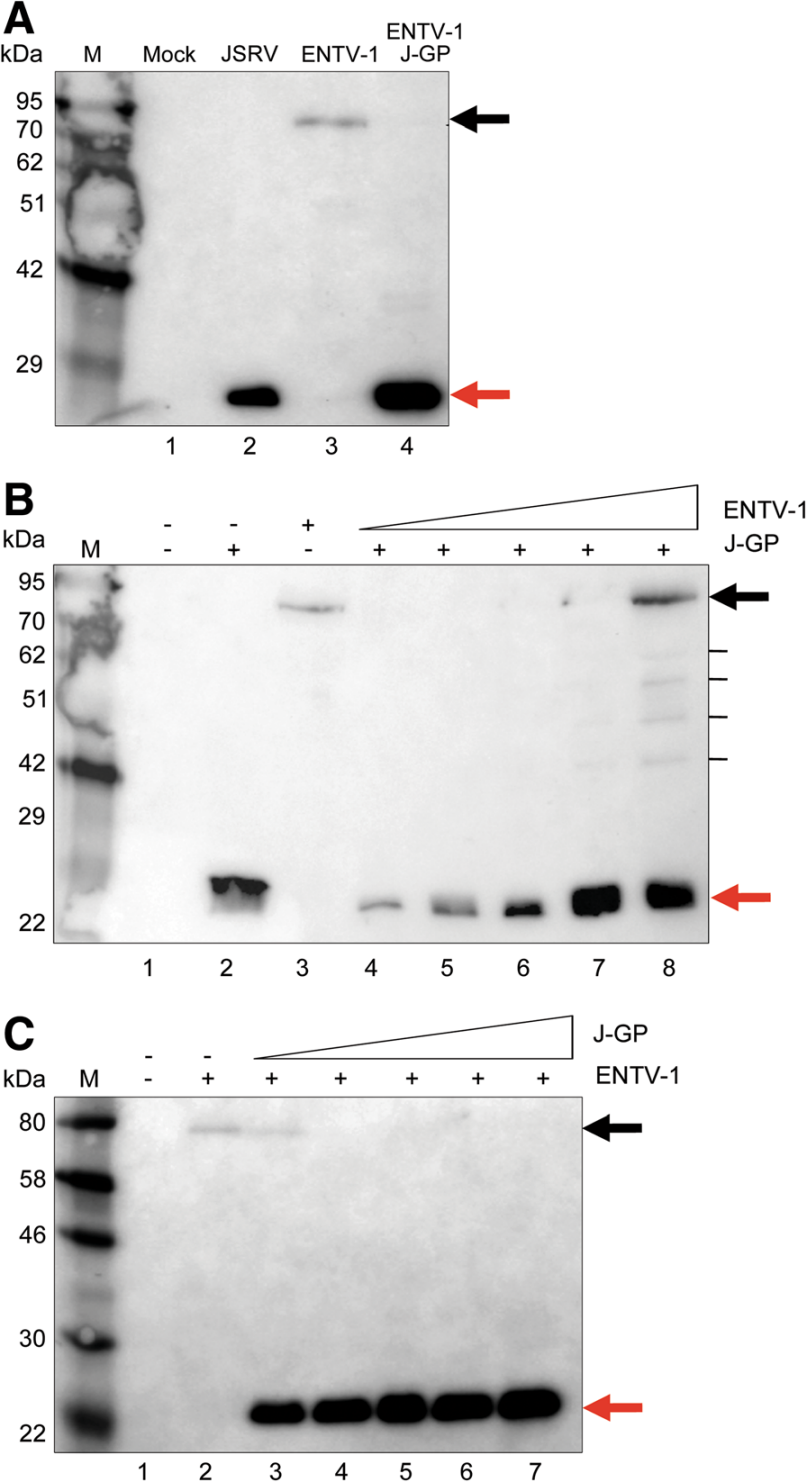
Dose dependent rescue of ENTV-1 polyprotein processing defect by complementation with JSRV Gag-Pro-Pol. **a** Capsid protein immunoblot analysis of virus particles obtained from HEK 293T cells co-transfected with the ENTV-1 molecular clone and the JSRV Gag-Pro-Pol expression vector (ENTV-1/J-GP) showing protease processing of the Gag polyprotein (lane 4). Virus derived from transfection of the JSRV (lane 2) and ENTV (lanes 3) molecular clones. The dosage dependence of polyprotein processing was evaluated by co-transfection of consistent amounts of JSRV Gag-Pro-Pol (0.5ug) with increasing amounts of ENTV-1 (1 to 10 μg) (**b**) as well as consistent amounts of ENTV-1 (5 μg) and increasing amounts of JSRV Gag-Pro-Pol (0.5 to 5 μg) (**c**). M indicates the lane containing the molecular weight marker. The *black arrows* indicate unprocessed Gag at 78 kDa and the *red arrows* indicate processed Capsid at 26 kDa

The mechanism responsible for initiating protease processing and maturation is not well understood. To determine whether the ENTV-1 genome and envelope protein contribute to the processing defect, the JSRV Gag-Pro-Pol polyprotein alone (pCMVGPP-MX-4CTE) was supplied in trans to allow co-assembly with the ENTV-1 polyprotein and packaging of the ENTV-1 genome thereby producing chimeric virus particles. HEK 293T cells were co-transfected with pCMVENTV-1 and pCMVGPP-MX-4CTE and virus purified from the supernatant was analyzed by Western blot using an antibody specific for the JSRV capsid (JCa). Virus from cells transfected with either the JSRV molecular clone (Fig. 2a, lane 2) or the ENTV-1 molecular clone (Fig. 2a, lane 3) showed banding patterns identical to those observed previously in Fig. 1. A single protein migrating at a molecular weight of 26 kDa was observed in virus particles produced from cells co-transfected with pCMVENTV-1 and pCMVGPP-MX-4CTE (Fig. 2a, lane 4) indicating that JSRV Gag-Pro-Pol can be packaged into the ENVT-1 virus and complement ENTV-1 processing defect. Since Gag polyprotein expression levels from the pCMVGPP-MX-4CTE plasmid were much greater than from either of the two molecular clones, we performed an experiment to determine the dose dependence of this complementation. Dose dependency experiments were performed with JSRV Gag-Pro-Pol maintained at a consistent level and ENTV-1 progressively increased (Fig. 2b) as well as the inverse (Fig. 2c). When ENTV-1 was supplied in excess, a band was detected at both 26 kDa and 78 kDa (Fig. 2b lane 7 and 8 and 2C lane 3). Proteins of approximately 62, 57, 52 and 40 kDa in a banding pattern reminiscent of partially processed Gag [14, 23] were also detected (Fig. 2b, lane 8). Since the JSRV Gag-Pro-Pol expression construct lacks a packaging signal, the virions produced in these experiments were presumed to have assembled around the ENTV-1 genome, indicating that the genomic RNA derived from the ENTV-1 molecular clone is not responsible for the processing defect and that the polyprotein cleavage sites are intact.

### Mutagenesis of the ENTV-1 protease to resemble the JSRV protease does not restore proteolytic processing

**Fig. 3:**
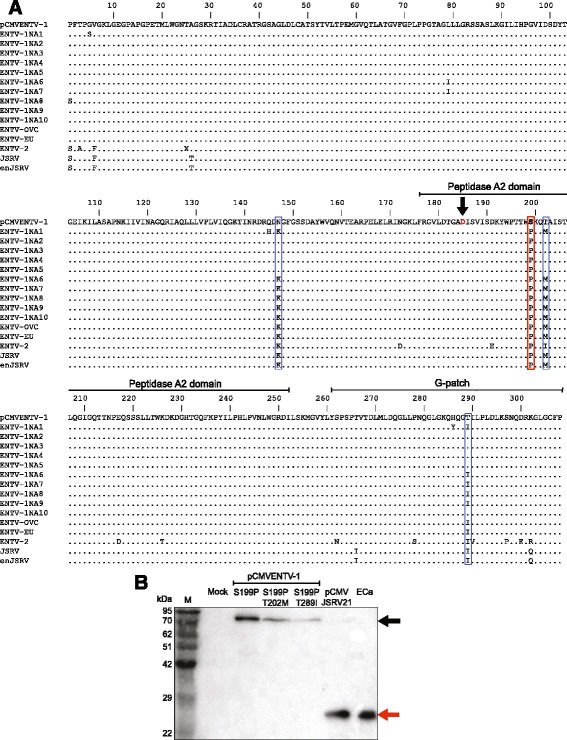
Mutagenesis of the ENTV-1 protease to resemble the JSRV protease does not restore proteolytic processing. **a** ClustalW alignment of the predicted amino acid sequence of the protease from the ENTV-1 molecular clone with that of all 10 ENTV-1 isolates from North America (ENTV-1NA, see Table 1 [19] for accession numbers), ENTV-1OVC (accession number KC189895) [41], ENTV-2 (accession number NC 004994), JSRV (accession number AF105220) and an endogenous JSRV (enJS56A1, accession number AF153615). *Dots* indicate identical amino acids. The *black arrow* marks the catalytic amino acid, shown in *red*. The *red box* highlights the S199P mutation found only in the original ENTV-1 molecular clone. The *blue boxes* highlight amino acids where the ENTV-1 molecular clone is more similar to ENTV-1NA4, from which it was derived, and the three other isolates that were obtained from the same farm as ENTV-1NA4: ENTV-1NA2, ENTV-1NA3, and ENTV-1NA5. **b** Capsid protein immunoblot analysis of lysates from concentrated viruses derived from the ENTV-1 molecular clone containing the designated amino acid changes in the protease domain. *M* indicates the lane containing the molecular weight marker and lanes labelled as ECa contain bacterially expressed and purified ENTV-1 capsid protein. The *black arrow* indicates unprocessed Gag at 78 kDa and the *red arrow* indicates processed Capsid at 26 kDa

**Fig. 4:**
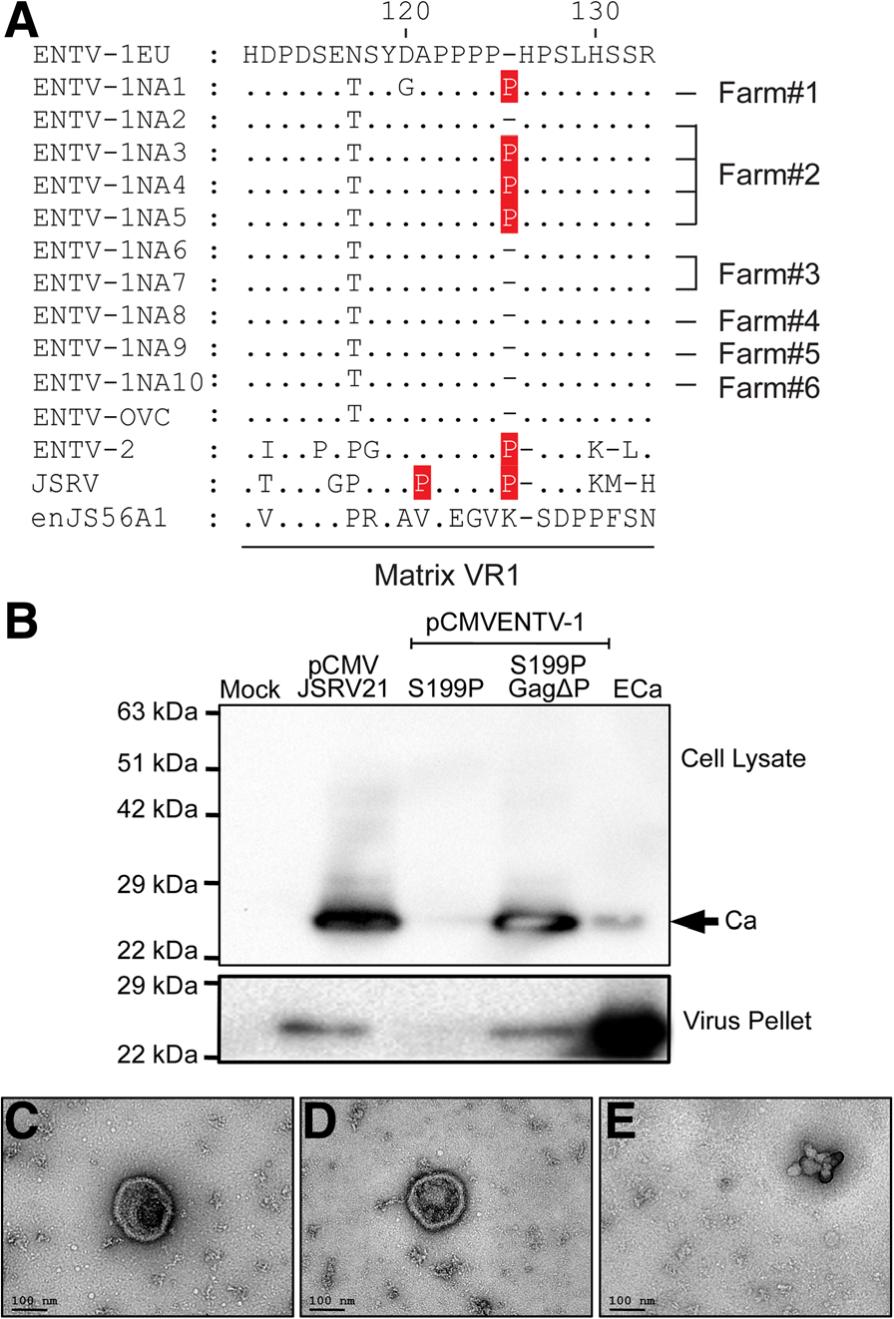
Deletion of a proline residue from the polyproline tract in the matrix protein results in production of fully processed ENTV-1 virions. **a** ClustalW alignment of variable region 1 (VR1) of the matrix protein from a European isolate of ENTV-1 (ENTV-1EU, accession number NC 007015), all ten ENTV-1 isolates from North America (ENTV-1NA, see Table 1 [19] for accession numbers), ENTV-2 (accession number NC 004994), JSRV (accession number AF105220) and an endogenous JSRV (enJS56A1, accession number AF153615). *Dots* indicate identical amino acids and dashes indicate deletions. Nonconserved proline residues are highlighted in *red*. **b** Immunoblot analysis of capsid in cell lysates or concentrated virus particles derived from the JSRV molecular clone (pCMVJSRV21), the ENTV-1 molecular clone with the S199P mutation (pCMVENTV-1S199P), and the final version of the ENTV-1 molecular clone with the S199P and the delta Pro mutation (pCMVENTV-1S199PGagΔP) after transfection of HEK 293 T cells. *M* indicates the lane containing the molecular weight marker and lanes labelled as ECa contain bacterially expressed and purified ENTV-1 capsid protein. **c** and **d** Two representative examples of transmission electron microscope analysis of recombinant ENTV-1 particles. **e** Transmission electron microscope analysis of control grid coated with concentrated cell-free supernatant derived from mock transfected cells

Since the ENTV-1 molecular clone was generated from two large overlapping PCR products, we investigated whether mutations might have been acquired during the amplification step, which could have affected protease activity. The ENTV-1 molecular clone, pCMVENTV-1, was sequenced in its entirety using primers described previously [19]. The protease sequence of pCMVENTV-1 was then compared to that of all North American ENTV-1 isolates, including the ENTV-1NA4 isolate from which pCMVENTV-1 was derived, the European ENTV-1 isolate, ENTV-2 from goats and finally, JSRV using MEGA5 software (Fig. 3a). A proline to serine change at position 199 unique to the pCMVENTV-1 protease was identified (outlined with a red box, Fig. 3a). This amino acid difference was located in the peptidase domain, 18 amino acids downstream from the catalytic aspartate residue, based upon sequence similarity and alignment with MPMV protease [24]. This mutation was of interest because prolines may influence secondary structure due to their “kinked” conformation [25, 26], and none of the available ovine betaretrovirus sequences possess a serine residue at this position. Two other sites of amino acid variability were noted, one in the peptidase domain (T versus M at position 202, outlined with a blue box in Fig. 4a) and the other in the G-patch domain (I versus T at position 289, outlined with a blue box highlighted in Fig. 3a). It should be noted that there was one additional amino acid difference that followed this pattern at position 147, but since it was localized to the dUTPase region of the polyprotein it was not included as a candidate for mutagenesis. Site-directed mutagenesis was performed on pCMVENTV-1 to first change the serine at position 199 to a proline (pCMVENTV-1S199P). Subsequent amino acid changes were introduced into pCMVENTV-1S199P to produce pCMVENTV-1S199P, T202M and pCMVENTV-1S199P, T289I, which resembled 9/15 of the ovine betaretrovirus sequences analyzed. Virus was generated from the modified molecular clones and analyzed by immunoblot for capsid protein expression (Fig. 3b). Despite mutating the protease of the ENTV-1 molecular clone to resemble that of known infectious viruses, there was no change in the processing defect of any of the mutant viruses as only unprocessed Gag could be detected (Fig. 3b). These results suggest that either all three amino acid variants within the protease need to be corrected or amino acid variants outside of the protease are contributing to the processing defect.

### Deletion of a proline residue from the polyproline tract in the matrix protein results in production of fully mature ENTV-1 virions

In searching for regions within Gag that might be contributing to the processing defect, it was curious that four of the ten full-length sequences we previously obtained for ENTV-1 isolates from North America possessed an additional proline residue in the polyproline tract of the matrix subunit of Gag (Fig. 4a), just upstream of a putative cleavage site [19]. We hypothesized that this additional proline could introduce a structural change in the protein that might reduce cleavage efficiency and/or alter the conformation of the protease such that it was no longer able to dimerize and become active. To investigate this possibility, the molecular clone containing the S199P correction (pCMVENTV-1S199P), was mutated to restore the four amino acid long polyproline tract in the Matrix protein. Indeed, the ENTV-1 molecular clone possessing a polyproline tract comprised of four consecutive proline residues (pCMVENTV-1-S199PGagΔP) resulted in the production of mature virions containing fully processed Gag (Fig. 4b). Transmission electron microscopy of a concentrated virus preparation detected spherical structures with an electron-dense, eccentric core, consistent with type B retroviruses (Fig. 4c, d), whereas only unstructured aggregates were found within the mock-transfected control (Fig. 4e). The average overall diameter of the structures (180 nm) is compatible with the predicted size of betaretrovirus particles [27].

## Discussion

ENA is a relatively common disease of sheep, particularly in North America [19, 28]. Despite this, little is known about the pathogenesis of the disease, including confirmation of ENTV-1 as the etiologic agent. Much of what is known about ENA and ENTV-1 is inferred from studies on OPA and JSRV. We endeavoured to address this problem and build upon our previous transmission experiments involving cell-free tumor homogenate by constructing a molecular clone of ENTV-1 from which virus could be generated for in vivo infection experiments. Failure of the original molecular clone to produce mature virions comprised of fully processed viral proteins presented a unique impediment to these experiments, but ultimately revealed important biological insights about ENTV-1.

Initially we thought that the defect in Gag polyprotein processing could be due to the requirement for a host cell protein found only in sheep cells or alternatively, due to the presence a restriction factor in human cells that inhibited maturation of ENTV-1 virions. To address this possibility, we attempted to produce recombinant ENTV-1 virions in both primary and continuous cell lines from sheep and goat; however, these cells were extremely resistant to transfection and did not yield sufficient amounts of virus for analysis, even after ultracentrifugation of virus-containing supernatant (data not shown). Indeed, it was difficult to observe ENTV-1 virions produced in HEK 293T cells in situations where the transfection efficiency was suboptimal; suggesting that virus production by this method is extremely inefficient. Since JSRV particles produced from HEK 293T cells are capable of polyprotein processing and the sequence identity between JSRV and ENTV-1 is relatively high, we concluded that it was unlikely that the defect in Gag polyprotein processing observed in virus particles produced from the ENTV-1 molecular clone was due to the absence of a critical host cell factor in the producer cells.

Co-transfection of the JSRV Gag-Pro-Pol expression vector with the ENTV-1 molecular clone generated virions with fully processed Gag (Fig. 2a). The antibody used in this experiment does not distinguish between JSRV and ENTV-1 capsid proteins so it was not possible to determine whether rescue of polyprotein processing was due to processing of ENTV-1 Gag by the JSRV protease or displacement of the ENTV-1 polyprotein by the JSRV polyprotein. The JSRV Gag-Pro-Pol expression vector contains additional elements (e.g., MPMV CTE and SV40 polyA) [17] that promote much higher expression of Gag-Pro-Pol than the molecular clone so direct comparisons are not possible. Expression level differences were taken into account in the dosage response co-transfection experiments (Fig. 2b and c) in order to encourage heterologous Gag polyprotein co-packaging (ie. generation of virions with polyproteins from both JSRV and ENTV-1) [29]. Incomplete rescue of processing, observed as partially processed Gag in lane 7 and 8 of Fig. 2b, indicates that the JSRV protease is processing the ENTV-1 polyprotein in trans in these virions but that there is a limit to which the JSRV protease can perform this function. Therefore, since the ENTV-1 polyproteins can be processed by the JSRV protease we concluded that the processing defect was due to a lack of ENTV-1 protease activity.

Interestingly, mutation of the pCMVENTV-1 protease to match that of ENTV-1NA4, the sequence from which it was derived, failed to rescue the processing defect. This was unexpected considering that the ENTV-1NA4 sequence was derived from nasal tumor tissue isolated from a sheep with ENA. Although only fully processed Gag was observed in virions purified from the ENTV-1NA4 tumor or any of the other ENTV-1NA tumors [14], a processing defect could exist outside the context of transformed sheep cells. Since ENTV-1OVC was extracted from a tumor transmitted by cell-free tumor homogenate [14], it was assumed that this sequence represented a replication-competent, infectious ENTV-1. It should be noted that homogenates from the ENTV-1OVC tumor contained unprocessed and partially processed Gag proteins which were thought to represent naked cores released from the cytoplasm of tumor cells due to mechanical disruption [14]. It is possible that the processing defect observed in virions derived from the initial ENTV-1 molecular clone may be reflective of the phenotype of at least some circulating exogenous ENTV-1 particles. If that were the case, these virions would not be infectious unless complemented with an active protease, perhaps from endogenous betaretrovirus sequences [30]. Expression of the endogenous ovine betaretroviruses is localized primarily to tissues of the reproductive tract [31]; however, transcripts have been detected in other tissues such as the lung [32]. Human endogenous retrovirus (HERV) sequences are upregulated in neoplastic tissues due to genomic instability and epigenetic changes during transformation [33–35] and a similar phenomenon occurs in HIV-infected cells [36]. Furthermore, HERV-K (a member of the *Betaretrovirus* genus) Gag can co-assemble and co-package with HIV-1 Gag [37]. Similarly, it has been shown that endogenous JSRV Gag can co-assemble with exogenous JSRV Gag and, in the presence of a particular mutation (R21W), restrict the release of exogenous JSRV particles in a transdominant fashion [38, 39]. The high degree of similarity shared between ENTV-1, JSRV and endogenous ovine betaretroviruses [19] suggests that since ENTV-1 can co-assemble with JSRV, it can likely also co-assemble with at least some of the endogenous ovine betaretrovirus Gag proteins. Therefore, it is conceivable that infection of sheep cells with ENTV-1 and subsequent transformation results in upregulation of endogenous betaretrovirus transcription, which in turn supplies polyproteins with active protease for co-packaging.

Since modification of the protease sequence of the defective molecular clone to match that of ENTV-1OVC failed to restore Gag polyprotein processing, we investigated sequence differences outside of the *pro* reading frame that might be responsible for the defect. In HIV, regions upstream of the protease play a critical role in protease activation by influencing dimerization of the protease domain in the precursor [40], which is requirement for activation [41]. Of the ten ENTV-1 genomes we previously sequenced, four possessed a polyproline motif comprised of five consecutive prolines within the matrix region of Gag, which differed from the four consecutive prolines that comprised this motif in the other six ENTV-1 genomes, as well as in the genome of an ENTV-1 virus isolated in the UK. Despite being located in one of three variable regions in the ovine betaretrovirus genome, this polyproline motif is conserved within virus families and is consistently comprised of seven consecutive prolines in the case of JSRV and six consecutive prolines in the case of ENTV-2. Interestingly, this polyproline motif is absent from endogenous versions of JSRV [42]. Since proline residues are known to introduce kinks in protein structures we theorized that the additional proline residue in the Gag-Pro-Pol polyprotein might prevent the protease from folding correctly, precluding dimerization and activation. Indeed, mutation of the molecular clone to restore the four amino acid long polyproline motif resulted in the recovery of mature virions containing fully processed Gag. Exactly why the addition of a proline residue to this motif had such a detrimental effect on polyprotein processing is not known; however, one might surmise that it could contribute to conformational changes in the polyprotein that alter protein-protein interactions, or as in the case of the Vpx protein of HIV-2, may reduce translation efficiency. Vpx proteins of the HIV-2/SIVsmm/SIVmac group encode a highly conserved polyproline motif (PPM) consisting of a hepta-proline stretch in the C-terminal region [43, 44]. Recently, the PPM in HIV-2 Vpx was shown to be critical for its efficient expression in both eukaryotic and prokaryotic systems and this effect is determined by the context of PPM amino acid sequences, not the nucleotide sequences [45]. Importantly, the number and position of consecutive prolines in this PPM were important for Vpx expression. PPMs are found in a large number of eukaryotic and prokaryotic proteins as well as in a wide range of human DNA and RNA viruses including herpesviruses, adenoviruses, and hepatitis viruses. However, whether these PPMs are responsible for efficient expression of the PPM-containing proteins or play some other as yet unidentified role in protein function is not yet known.

## Conclusions

In summary, we have constructed a molecular clone of ENTV-1 that is capable of directing the production of mature virus particles containing fully processed Gag-Pol. As there are no cell lines that support replication of ENTV-1, experiments are underway to confirm the infectious nature of virus produced from this molecular clone in newborn lambs. Construction of an ENTV-1 molecular clone will allow for the first time detailed investigations into the mechanisms contributing to the pathogenesis and tissue tropism of this poorly understood virus.
